# Fetal MRI findings in conjoined twin pregnancies

**DOI:** 10.1007/s00247-025-06323-1

**Published:** 2025-08-15

**Authors:** Julie E. Walcutt, Beth M. Kline-Fath, Rama S. Ayyala, Foong-Yen Lim, Usha D. Nagaraj

**Affiliations:** 1 Children’s Nebraska, 8200 Dodge St, Omaha, NE 68114 USA; 2 University of Nebraska Medical Center, Omaha, USA; 3 Cincinnati Children’s Hospital Medical Center, University of Cincinnati, Department of Radiology, 3333 Burnet Avenue, MLC 5031, Cincinnati, OH 45229 USA; 4 University of Cincinnati Medical Center, Cincinnati, USA

**Keywords:** Pregnancy, Twins, Monozygotic, Twins, Conjoined, Pregnancy, Twin, Prenatal care, Magnetic resonance imaging

## Abstract

Conjoined twins are a rare and complex anomaly of monozygotic twin gestations, in which there is joining of the bodies of two individuals. Prenatal imaging, including fetal MRI, plays a crucial role in prognostication and planning for these twins’ families and multi-disciplinary medical teams. The ability of fetal MRI to provide large field-of-view imaging is well-suited to delineate the complicated shared and individual anatomy of these twins. In this review, we describe an approach to the fetal MRI of conjoined twins and its limitations, illustrate the fetal MRI findings of the conjoined twin morphological variants, and discuss common superimposed anomalies. The anatomic features most predictive of these twins’ chances for survival and separation are also summarized.

**Supplementary Information:**

The online version contains supplementary material available at 10.1007/s00247-025-06323-1.

## Introduction

Conjoined twins are an extraordinary and puzzling complication of monozygotic twin gestations, in which there is joining of the bodies of two individuals. Ultimately, the mechanism by which conjoined twinning occurs remains unknown [1]. There is no apparent genetic basis for the development of conjoined twins; in one study, all cases with analyzed karyotypes were normal [2]. The contradictory fission and fusion theories have been proposed as mechanisms for the etiology of conjoined twins. The fission theory states that conjoined twins are the result of the division of a single embryo after post-fertilization day 13–14, a mechanism which purportedly accounts for the mirror imaging anatomy and laterality errors seen in conjoined twins [3]. Arguing that there is no known mechanism by which a vertebrate embryo could be made to divide, the proponents of the competing fusion theory propose that conjoined twins arise from the fusion of two formerly separate embryos at either the yolk sac or the neural tube, which lack intact ectoderm [4]. Despite their uncertain origins, conjoined twins are an undisputably rare occurrence. One international epidemiological study found a total prevalence of conjoined twins of 1.47 per 100,000 births, although the rates varied by the reporting sites [2]. Conjoined twins are more commonly female, with a ratio of roughly 2:1 or greater [2, 5–9].

Beyond their naturally low incidence, the high mortality rates for conjoined twins make those pairs surviving long-term truly exceptional. An international study of conjoined twin births between 1968 and 2006 reported a live-birth rate of 46% with the remaining pregnancies divided equally between stillbirths and elective terminations (27% each) [2]. A study of conjoined twins born in the USA between 1997 and 2012 found only 39% of individuals were discharged alive, with most deaths occurring within the first 24 h of life [7]. Globally, as few as 6–8 pairs of conjoined twins each year are estimated to undergo surgery as infants [10].

Given the high stillbirth rate and perinatal mortality of conjoined twins, fetal imagers are uniquely positioned to encounter the full morphological spectrum of conjoined twins. Prenatal imaging of these complex patients provides essential information to guide prognostic counseling and plan for their postnatal medical and surgical needs [11, 12]. The capability of fetal MRI to provide large field-of-view images is ideally suited to depicting the unique and often complicated anatomy of conjoined twins and is especially helpful when fetal ultrasound is limited by fetal position, maternal body habitus, or oligohydramnios [13, 14]. Fetal MRI may be of particular value if emergency separation shortly after birth may be required prior to comprehensive postnatal imaging [8, 15].

This review aims to describe the morphological variants of conjoined twins, the patterns of organ sharing and associated anomalies, and their implications for survival, morbidity, and potential separation. We highlight the role of fetal MRI in assessing these structural findings, and its contribution to prenatal evaluation and surgical planning.

## Approach to and limitations of fetal MRI in conjoined twin pregnancies

**Fig. 1:**
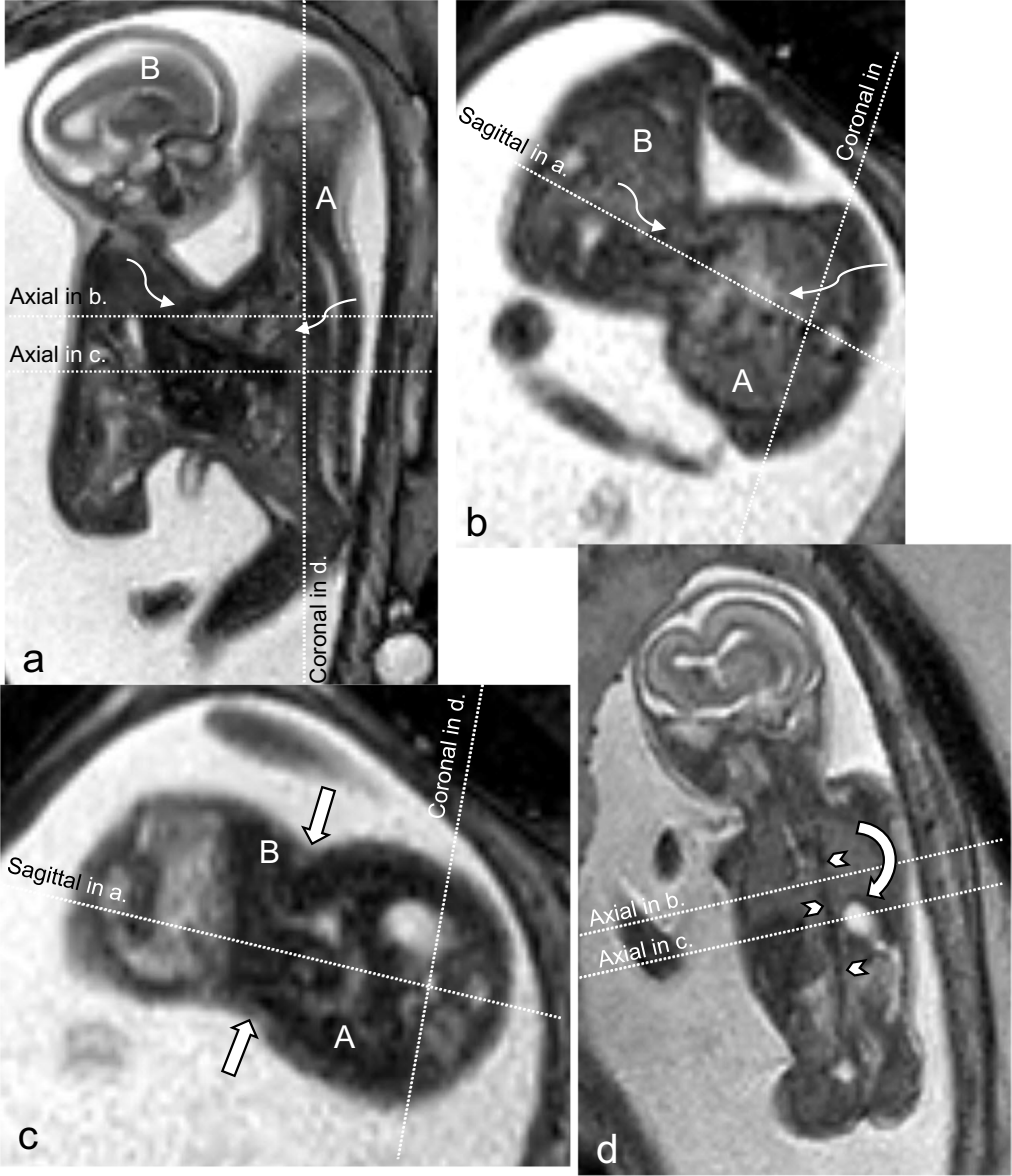
Fetal MRI of thoracopagus at gestational age 20 weeks, 4 days, demonstrating imaging planes. *Dashed lines* are labeled to indicate the approximate orthogonal imaging planes depicted in the other figure parts. **a** BTFE image sagittal to the ventrally conjoined torsos of the twins. This imaging plane is also sagittal to the brain of twin B. Twin A’s head is turned out of the imaging plane. Dedicated imaging of the twins’ brains required individualized imaging planes (*not shown*). Much of the shared heart (*between thin curvy white arrows*) is positioned within the chest of twin A, a feature that helps to differentiate the twins. **b** BTFE image axial to the shared chest. Axial plane of imaging redemonstrates the shared heart eccentrically positioned within twin A (*between thin curvy white arrows*). **c** BTFE image axial to the asymmetric shared abdomen. Twin A’s abdomen is larger and contains more of the shared liver (*between white arrows*) than twin B’s abdomen. **d** T2 SSH coronal to twin A. Left-sided aorta (*white arrowheads*) and stomach (*curved white arrow*) demonstrated. *BTFE*, balanced turbo-field echo; *SSH TSE*, single-shot turbo spin-echo

As in the MRI evaluation of fetal singletons, a combination of sequences is used when imaging conjoined twins, including single-shot fast spin-echo T2 or half-Fourier single-shot turbo spin-echo for organ detail, balanced steady-state free precession for bright-blood analysis of the vasculature, T1-weighted imaging for depiction of meconium-filled bowel, diffusion-weighted imaging for brain sharing and kidney localization, and echo-planar black bone imaging for evaluation of the skeletal structures [16]. Polyhydramnios is observed in approximately 50% of conjoined twin pregnancies and may impact imaging quality [14]. Imaging planes generally include axial, sagittal, and coronal assessments of the twins’ torsos and brains (Fig. 1). Depending on the twins’ morphological subtype, the imaging planes may need to be individually prescribed for each twin to optimize evaluation of their separate anatomy. Reference to a dictation template may serve as a helpful reminder while prescribing the scan to ensure all structures are adequately imaged and will later facilitate reporting (Supplementary Table 1). Naming assignments of the twins should attempt to follow that of previous imaging [16]. Anomalies present in only one twin or asymmetries at their site of union are helpful in differentiating between the twins.

Like fetal ultrasound, fetal MRI has its own limitations. For conjoined twins with liver sharing, fetal MRI may not be able to adequately delineate whether the biliary anatomy is separate or shared, an important consideration for separation. Similarly, fetal MRI may not be able to resolve potentially complex vascular sharing. In conjoined twins with a shared peritoneal cavity and intermingling of bowel loops, fetal MRI will usually not be able to determine whether the small bowel is shared, and this may be unknown until the time of surgery, as postnatal enteral contrast studies may be similarly limited [17]. Small fetal size or persistent motion at the time of fetal MRI will further limit evaluation of all structures. Postnatal contrast-enhanced MRI, CT, fluoroscopic studies, and 3D reconstructions will most likely be required to further characterize the anatomy outlined by fetal MRI [18].

## Morphological variants of conjoined twins

### Terminology

“Symmetric” conjoined twins, in which each twin of the pair is similarly developed and homologously united to their co-twin, are the topic of this review. “Parasitic” conjoined twins, in which the anatomical components of a nonviable “parasitic” twin are variably conjoined to a more typically developed co-twin, are not discussed [4]. Symmetric conjoined twins generally demonstrate classifiable patterns of union. Such union is homologous, meaning the twins are joined at the same parts and in generally the same orientation [4]. Each of these morphological variants is named according to the specifics of their union with the suffix “-pagus,” meaning “fixed” in Greek [4]. This review will use a classification scheme proposed by Spencer, which broadly groups the conjoined twin morphological variants by the primary orientation of their union, either ventral or dorsal, with laterally conjoined twins considered to be a variant form of ventral union [4, 19, 20]. The ventrally conjoined morphological variants include thoracopagus, omphalopagus, cephalopagus, ischiopagus, and the ventrolaterally conjoined parapagus. The dorsally conjoined morphological variants include craniopagus, pygopagus, and rachipagus [19]. In general, ventrally (and laterally) conjoined twins will share an umbilicus, and dorsally conjoined twins will not [20]. Shared umbilical cords may have two to six vessels, but often have fewer than six vessels [21].

Not all conjoined twin pairs will align perfectly with these described morphological variants, but because pairs within a morphological category share patterns of organ union and anomalies, use of this terminology facilitates communication with the clinical team, which can begin to anticipate the challenges of survival and separation of an individual pair. Ultimately, however, the details of their unique anatomy will be of greater importance [18]. It is important to note how authors have defined the morphological variants of their conjoined twin cohorts when utilizing these references for prognostication, especially with regard to categorization of pairs as thoracopagus versus omphalopagus. The defining characteristics and frequencies of the conjoined twin morphological variants are described and illustrated in Table 1.

**Table 1:**
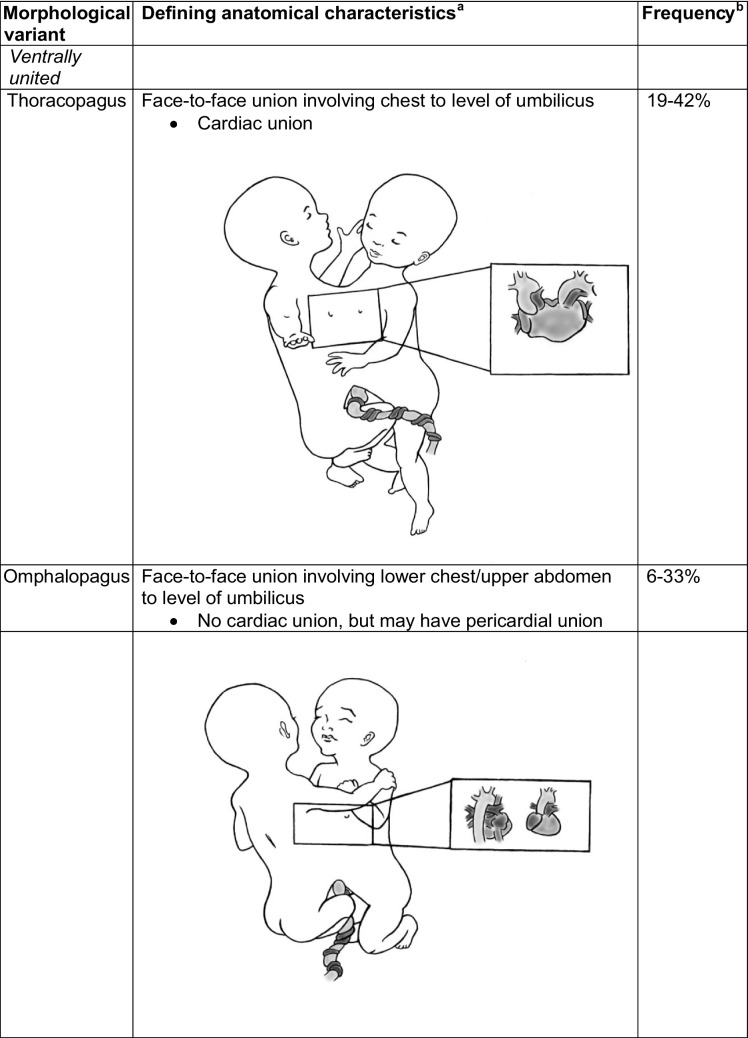

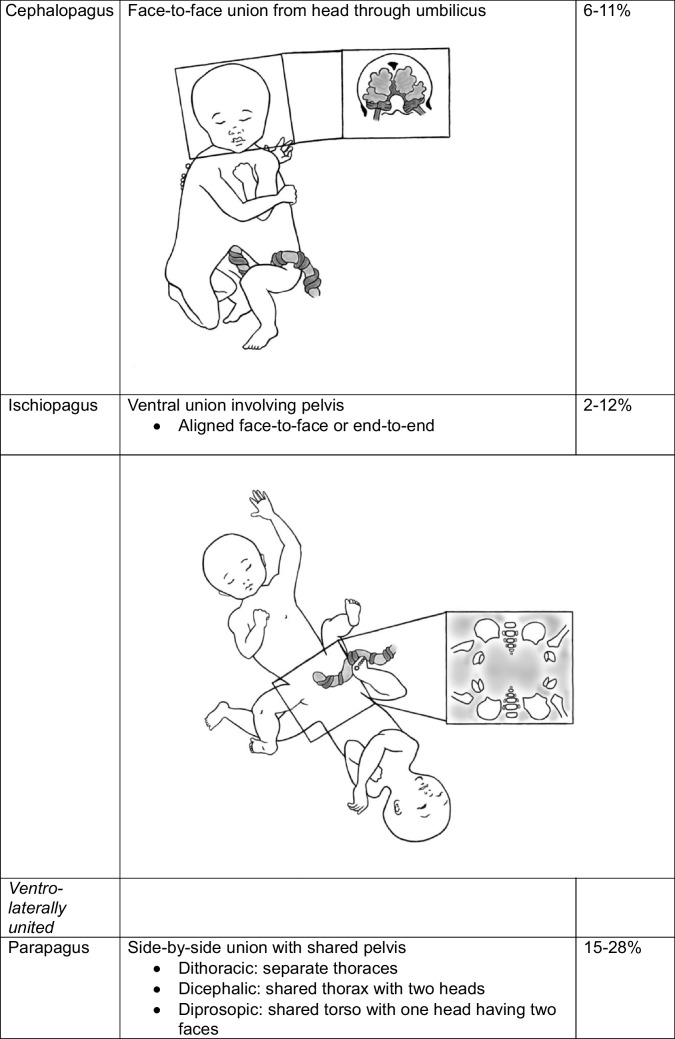

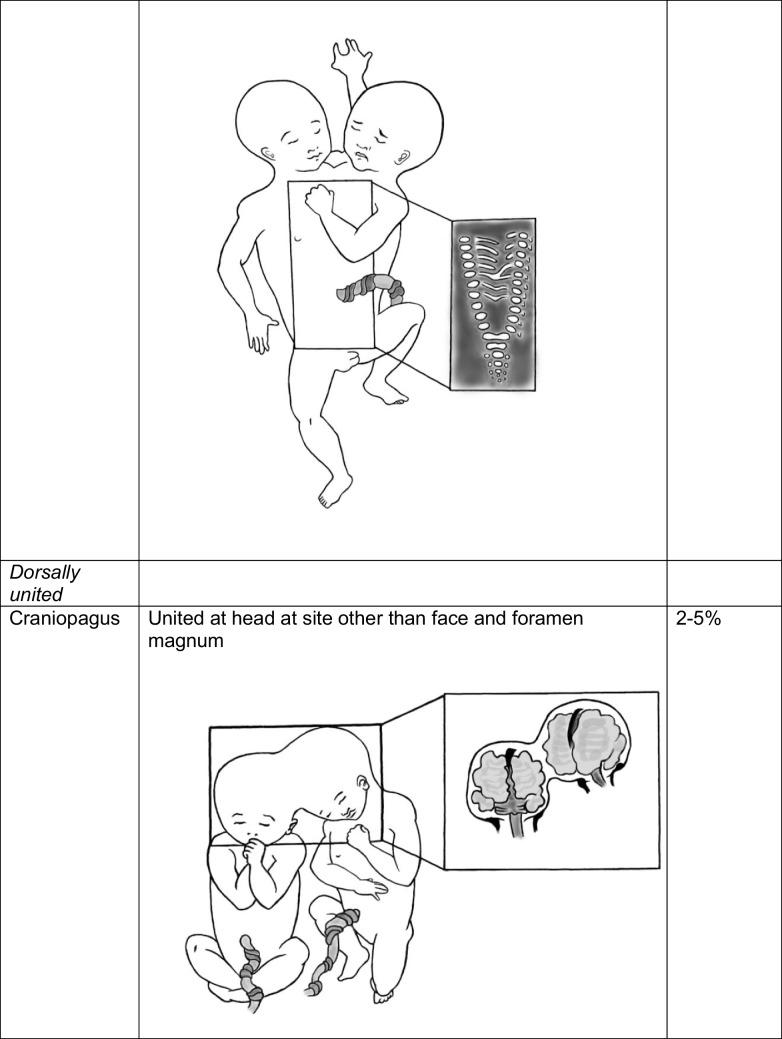

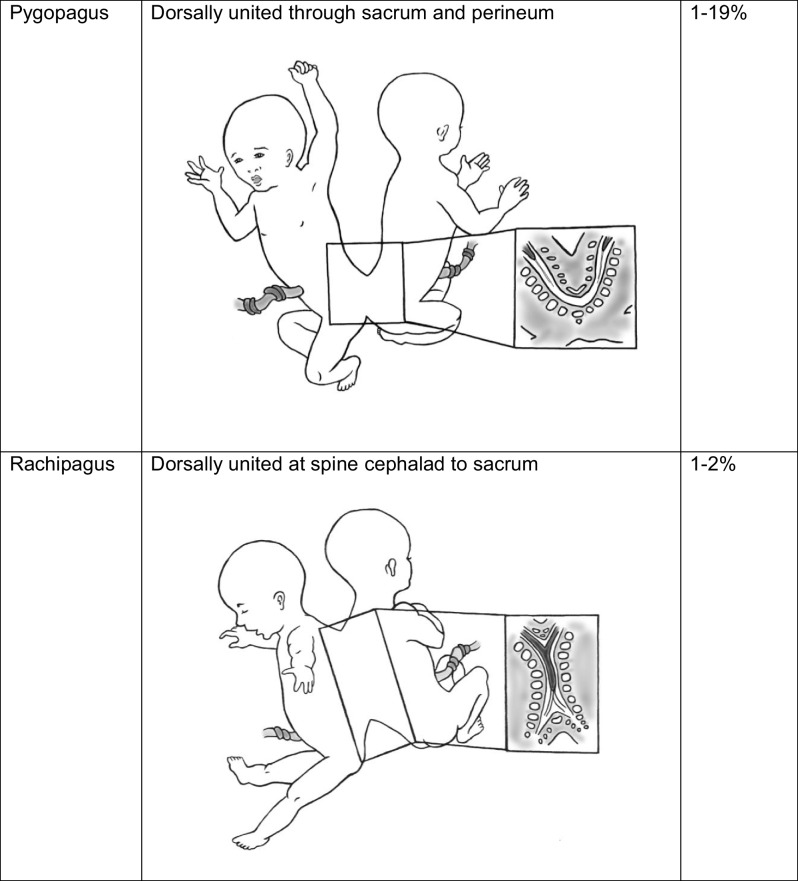
Defining characteristics and frequencies of conjoined twin morphological variants

^a^[19]

^b^[2, 4, 22, 23]

Original artwork by Julie Walcutt, edited with Photoshop 2025

#### Ventrally conjoined twins

##### Thoracopagus

**Fig. 2:**
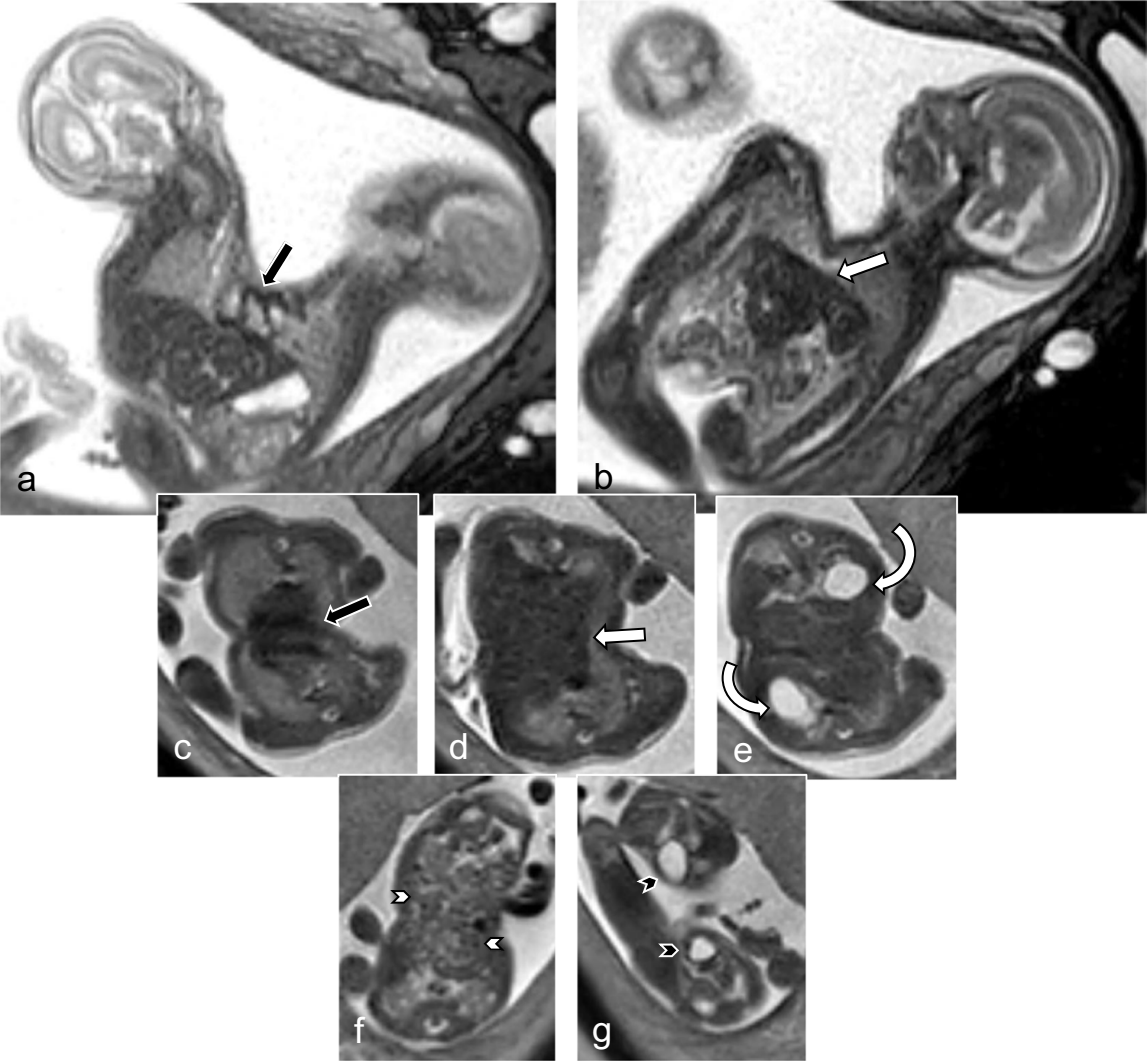
Fetal MRI of thoracopagus at gestational age 20 weeks, 5 days, showing ventral fusion of the chest and abdomen. **a**-**b** BTFE sagittal images of the ventrally conjoined chest and abdomen show a shared multi-chamber heart (*black arrow*) and shared liver (*white arrow*). **c**-**f** T2-weighted SSH TSE sequential axial images of the shared chest and abdomen show the shared heart (*black arrow*) with separate lungs, shared liver (*white arrow*), separate stomachs (*white curved arrows*), and shared peritoneal cavity with bowel (*white arrowheads*). **g** T2-weighted SSH TSE axial image through separate pelvises shows separate urinary bladders (*black arrowheads*). *BTFE*, balanced turbo-field echo; *SSH TSE*, single-shot turbo spin-echo

Thoracopagus is the most common conjoined twin morphological variant reported in most series [2, 4, 22, 23]. Thoracopagus are joined ventrally, face-to-face, from the chest through the umbilicus with four arms and four legs [19] (Fig. 2). According to the classification described by Spencer, this union always involves the heart, though the degree of cardiac sharing may range from a single patent vessel to a single heart perfusing both twins [21]. The shared heart may be multichambered with associated anomalous vessels [24]. Importantly, not all authors have strictly adhered to Spencer’s definition when categorizing their conjoined twin pairs. Some reports of thoracopagus have included pairs with only shared pericardium but separate hearts, or pairs with chest fusion but separate hearts and pericardia, which may be better classified as omphalopagus (described below) [25–27]. Although the diaphragm may be fused, lung fusion is uncommon among thoracopagus [1, 21, 28]. However, lung volumes between the twins may be asymmetric, and pulmonary hypoplasia may result from enlargement or displacement of the shared heart [11]. The liver is essentially always shared by thoracopagus with approximately 17–25% sharing biliary systems [4, 20, 23–25, 29, 30]. Even when two separate gallbladders are present, there may be fusion of the extrahepatic biliary systems. About half of thoracopagus have shared small bowel that typically converges in the duodenum and diverges at the level of a Meckel’s diverticulum [31–33].

##### Omphalopagus

**Fig. 3:**
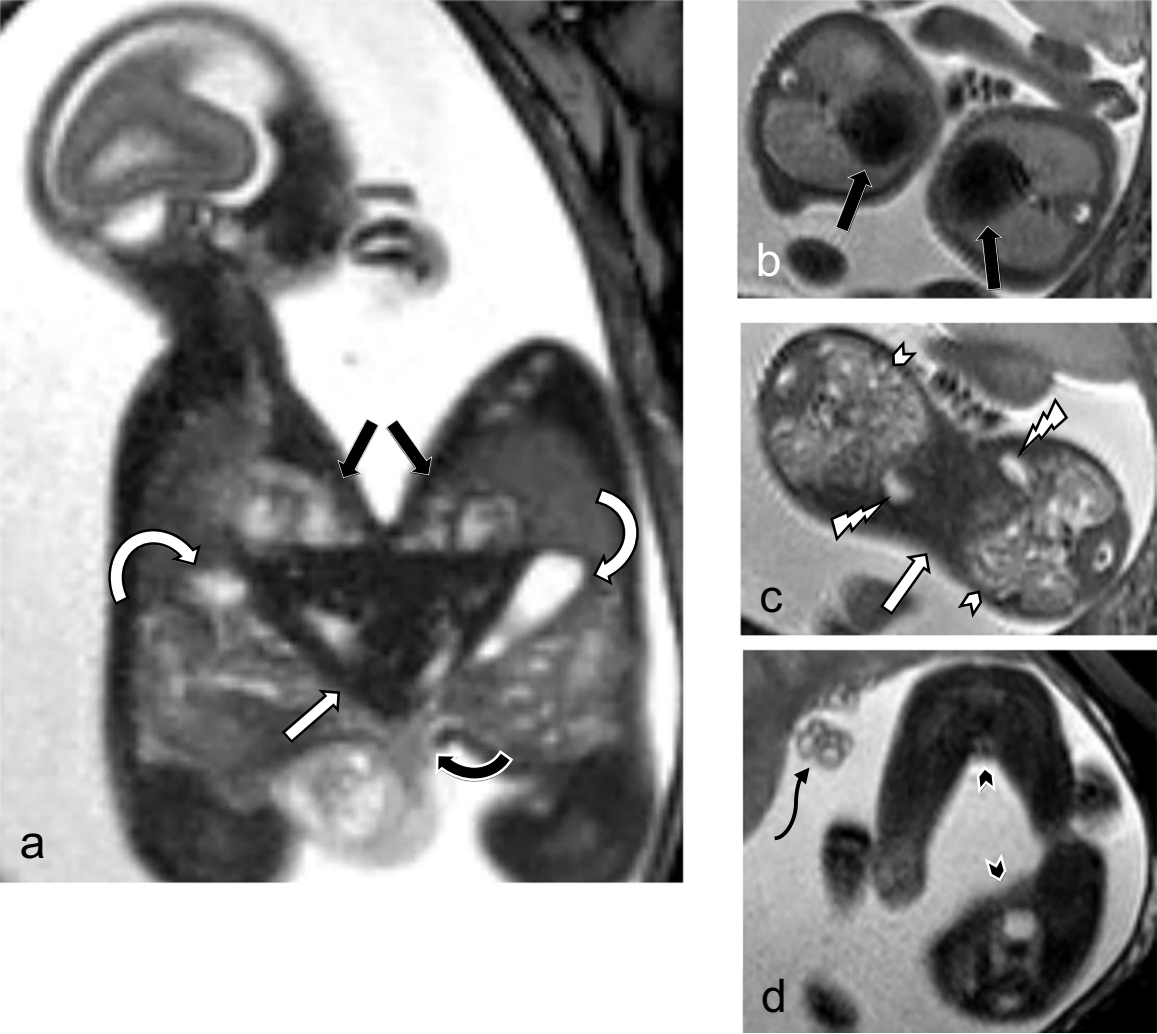
Fetal MRI of omphalopagus at gestational age 21 weeks, 3 days, showing ventral fusion of the lower chest and abdomen. **a** BTFE sagittal image shows separate hearts (*black arrows*), separate stomachs (*white curved arrows*), shared liver (*white straight arrow*), and shared umbilical cord abdominal insertion (*curved black arrow*). **b**-**c** T2-weighted SSH TSE sequential axial images through the torso show separate hearts (*black arrows*), shared liver (*white arrow*), separate gallbladders (*white lightning bolts*), and separation of the bowel (*white arrowheads*). **d** BTFE axial image shows separate pelvises (*black arrowheads*). Multivessel umbilical cord also seen (*thin curvy black arrow*). *BTFE*, balanced turbo-field echo; *SSH TSE*, single-shot turbo spin-echo

Like thoracopagus, omphalopagus are joined ventrally, face-to-face, from the chest through the umbilicus with four arms and four legs [19]. However, by Spencer’s definition, omphalopagus are distinguished from thoracopagus by the presence of separate hearts, although there may be pericardial fusion or a solid fibrous cord [20, 21] (Fig. 3). Similar to thoracopagus, there may be diaphragmatic fusion [9]. Unlike ischiopagus (described below), omphalopagus have separate pelvises. Eighty percent of omphalopagus have a shared liver; however, fusion of the biliary system is uncommon [9, 20, 23]. Though omphalopagus ordinarily have separate stomachs and proximal small bowel, one-third may have bowel fusion at a Meckel’s diverticulum, resulting in a shared ileocolic segment upstream from separate rectums [14, 23]. Even if the intestines are separate, the bowel may cross midline within a shared peritoneal cavity [34]. Urachal structures may connect the twins’ bladders, and there may be a shared omphalocele [25]. Typically, however, the genitourinary systems of omphalopagus are separate [14]. In some cases, omphalopagus are joined only by a skin bridge [9]. If the point of conjunction is unusually pliable, one twin may transiently twist about this pedicle so as to assume a position inverse to that of their co-twin [21].

##### Cephalopagus

**Fig. 4:**
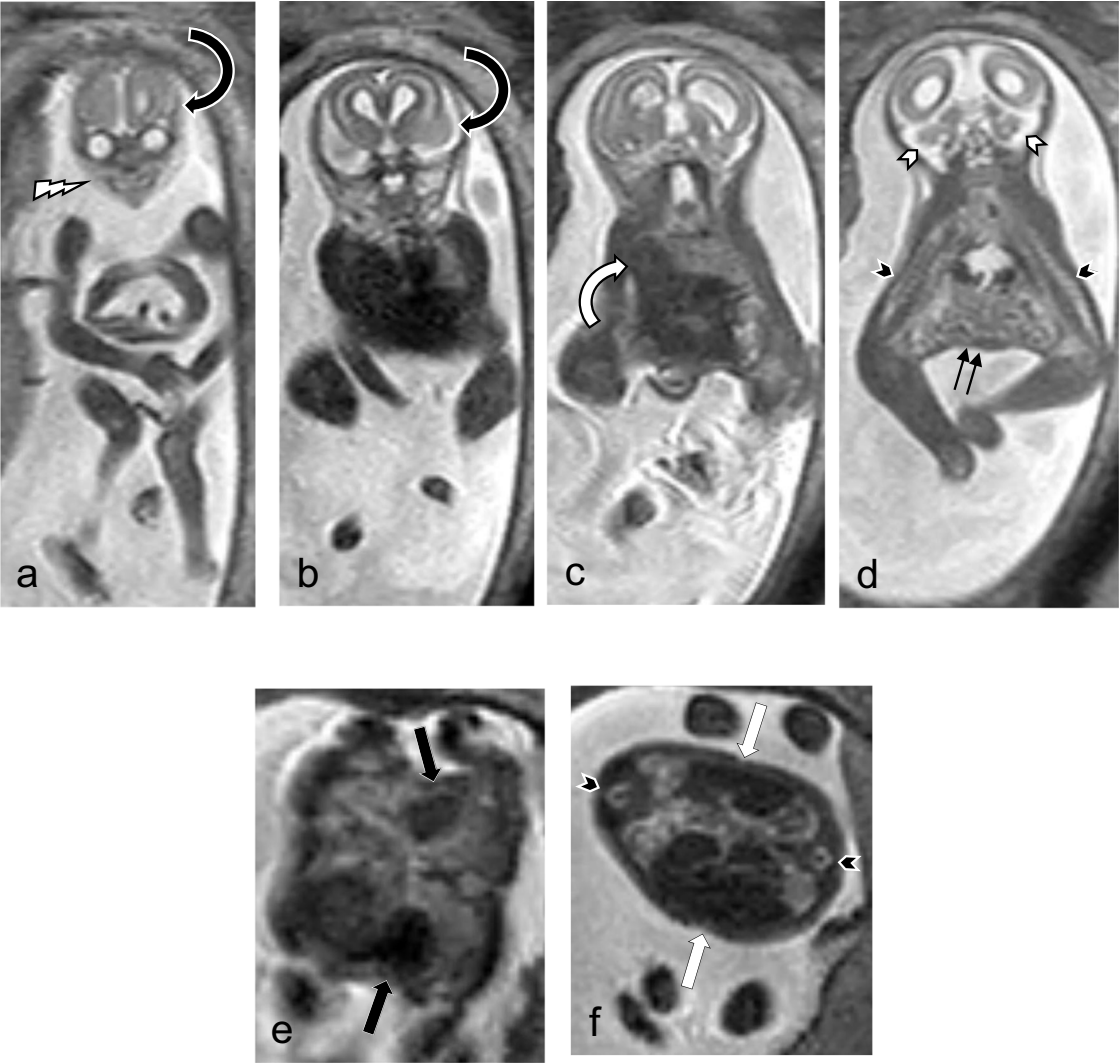
Fetal MRI of cephalopagus at gestational age 18 weeks, 3 days, showing ventral fusion of the head through the abdomen. **a**-**d** T2-weighted SSH TSE sequential ventral-to-dorsal coronal images show fused anterior brain (*curved black arrow*), single face (*white lightning bolt*), herniation of one liver through a congenital diaphragmatic hernia (*curved white arrow*), separate cerebellums (*white arrowheads*) and spinal canals (*black arrowheads*), and bowel in a shared peritoneal cavity (*double small black arrows*). **e** T2-weighted SSH TSE axial image through the conjoined chest shows separate hearts (*black arrows*). **f** T2-weighted SSH TSE axial image through the conjoined abdomen shows separate livers (*white arrows*) and spinal canals (*black arrowheads*). *SSH TSE*, single-shot turbo spin-echo

Like thoracopagus (and some omphalopagus), cephalopagus are ventrally joined from the chest through the umbilicus with four arms and four legs [19, 35]. However, as their name suggests, cephalopagus have a shared head (Fig. 4). The two faces may be positioned next to one another on the same side of the shared head; varying degrees of fusion between the side-by-side faces may be seen, which, when extreme, may appear as a single fused face. Alternatively, the two faces may be positioned on opposite sides of the shared head (with half of each face belonging to each twin); these faces may be well-developed and symmetric or markedly asymmetric to the extreme that one face only consists of a small ectopically positioned proboscis. The cerebrum may demonstrate degrees of fusion, but the hindbrain and spinal cords are typically separate. Anencephaly and cleft palate have been observed in cephalopagus. The thoracic and abdominal viscera may be shared or duplicated. The duplicated organs may appear to correspond to an individual twin (such as the more posteriorly located pancreas, spleen, and kidneys), or they may have a stacked anterior–posterior relationship preventing this assignment, which has been described for the lungs and liver, although the liver is often fused in cephalopagus [20, 35]. There may be a single perfusing heart, potentially paired with a rudimentary heart, although two symmetrical hearts have been described [36]. Even when there are two mouths, fusion of the pharynx or esophagus and the downstream midgut is typically observed, with separation of the gut occurring in the jejunum or ileum at a Meckel’s diverticulum. Separate biliary tracts inserting onto a shared duodenum have been described. The pelvises, urogenital structures, and hindguts are separate [35].

##### Ischiopagus

**Fig. 5:**
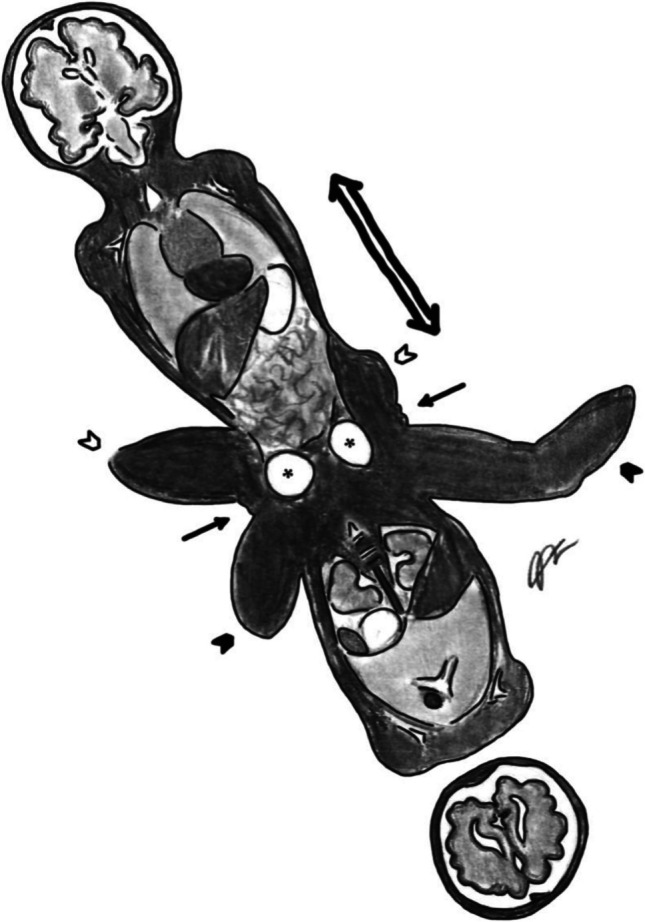
Artist’s representation of fetal MRI appearance of ischiopagus conjoined twins. Twins are joined at the level of the pelvis, arranged end-to-end along the axes of their separate spines (*open double-headed arrow*). There are four legs (out of the simulated imaging plane to varying degrees) with one leg on either side of the conjoined pelvis belonging to each twin (*open arrowheads* indicate upper twin’s legs and *black arrowheads* indicate lower twin’s legs). There are two sets of female external genitalia shared by both twins, positioned along the lateral margins of the conjoined pelvis between the two sets of shared legs (*black arrows*). Two side-by-side urinary bladders are present within the conjoined pelvis (*asterisks*). Four arms (two for each twin) and rectums are out of the simulated imaging plane. The remaining structures of the abdomen and chest are separate. Original artwork by Julie Walcutt, edited with Photoshop 2025

Unlike the other ventrally conjoined morphological variants, which demonstrate fusion above the level of the umbilicus, ischiopagus are united from the umbilicus through the pelvis [19]. These twins share a pelvis with two sacrums, typically without spinal fusion, although spinal cord fusion has been described [14, 31, 37]. The large conjoined pelvis usually has two laterally positioned pubic symphyses, to which each twin contributes a pubic ramus [21]. These twins have four arms, but they may have three or four legs [19, 25]. Ischiopagus take two typical forms. In the more common variant, the twins are joined end-to-end with the spines aligned on a common axis (Fig. 5). Less commonly, the twins are joined face-to-face with fusion of the infraumbilical abdomen and pelvis [19]. These twins have separate hearts [21]. With substantial ventral union, there may be liver fusion, although two portal triads are usually found [17, 20]. However, twins with extensive ventral fusion and fewer than four legs may be more accurately classified as parapagus (described below) [19]. One-half of ischiopagus have genitourinary tract fusion [29]. Within their shared pelvis, ischiopagus may have one or two bladders that may be side-by-side or midline in position, potentially with one bladder draining into the other [14, 38]. Even if there are two bladders, each bladder may drain ureters from both twins [14, 38, 39]. The urethra may be partially duplicated but typically drains to a shared urethral meatus [14, 31, 39]. Renal ectopia, malrotation, or fusion may be observed [14, 31]. Seventy percent of ischiopagus share their distal gastrointestinal tract, which may fuse at the level of a Meckel’s diverticulum [11, 24, 33, 38]. Anal atresia, colovesical fistulas, and cloacal anomalies have been encountered in ischiopagus [11, 14, 30, 38]. Both male and female ischiopagus often have four gonads, and females typically have duplicated uteri and external genitalia [25]. The two sets of external genitalia are each positioned laterally between the twins, corresponding to the location of the laterally displaced shared pubic symphyses and located between two legs, with one leg belonging to each twin [21]. Male ischiopagus have increased rates of undescended testes [14]. Ischiopagus may have hemivertebra, segmentation anomalies, and scoliosis [40].

#### Laterally conjoined twins

##### Parapagus

**Fig. 6:**
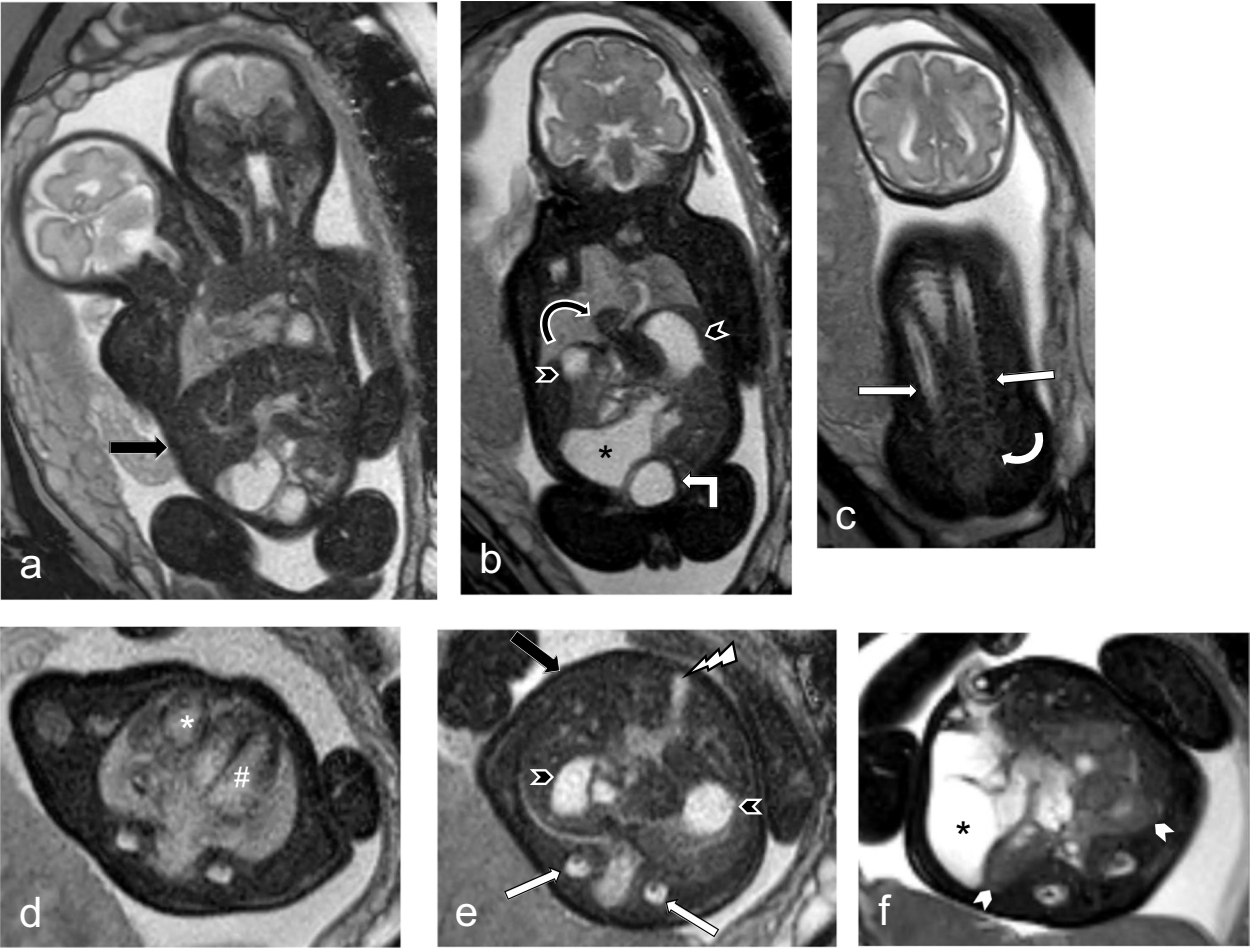
Fetal MRI of dicephalic parapagus at gestational age 32 weeks, 1 day, showing lateral fusion of the torso with separate heads. **a**-**c** BTFE sequential ventral-to-dorsal coronal images show the shared liver (*black arrow*), separate stomachs (*black arrowheads*), single urinary bladder (*white right-angle arrow*), and two spinal canals (*white arrows*) with osseous fusion at the level of the pelvis (*curved white arrow*). There is also cephalad protrusion of a small midline component of the shared liver representing a small diaphragmatic hernia or eventration (*curved black arrow*) and a dilated fluid-filled loop of bowel in the right lower quadrant (*black asterisk*), raising concern for bowel atresia or midgut volvulus. **d** BTFE axial image through the shared chest shows two asymmetric side-by-side hearts (*white asterisk* and *white pound sign*). The smaller right-sided heart (*white asterisk*) was shown to have a single ventricle on fetal echocardiography. **e** BTFE axial image through the upper abdomen shows the shared liver (*black arrow*), single gallbladder (*white lightning bolt*), separate stomachs (*black arrowheads*), and two spinal canals (*white arrows*). **f** BTFE axial image through the lower abdomen shows two kidneys (*white arrowheads*) and the dilated bowel loop (*black asterisk*) also depicted in **b**. *BTFE*, balanced turbo-field echo

Parapagus, like ischiopagus, share a pelvis. However, the parapagus union is lateral, with the twins positioned side-to-side. Parapagus typically share a pubic symphysis but may have one or two sacrums. They may have two or three legs and two, three, or four arms. The degree of fusion above the level of the pelvis is variable. Dithoracic parapagus have separate thoraces. Dicephalic parapagus have a single shared thorax and two separate heads (Fig. 6). Diprosopic parapagus share a torso and a head with two faces, which are on the same side of the shared head [19]. Anencephaly may occur in as many as two thirds of diprosopic parapagus [20]. Parapagus fused through the chest may have separate or shared hearts; if shared, the heart may have the anomalies typical of singletons or may be multichambered [14, 20]. Eighty percent of dithoracic parapagus have separate hearts, although frequently found to be abnormal [41]. Essentially all parapagus have a shared liver [4, 20, 21]. While diprosopic parapagus share their entire intestinal tract, other less extensively fused forms of parapagus typically share colon and rectum downstream from a Meckel’s diverticulum, possibly complicated by colovesical fistula or anal atresia [17, 20, 31]. Parapagus may occasionally have two anuses [17, 21]. Genitourinary anomalies of the shared pelvis may be observed [31]. Interestingly, anomalies have been more commonly observed in the right-sided twin [19].

#### Dorsally conjoined twins

##### Craniopagus

**Fig. 7:**
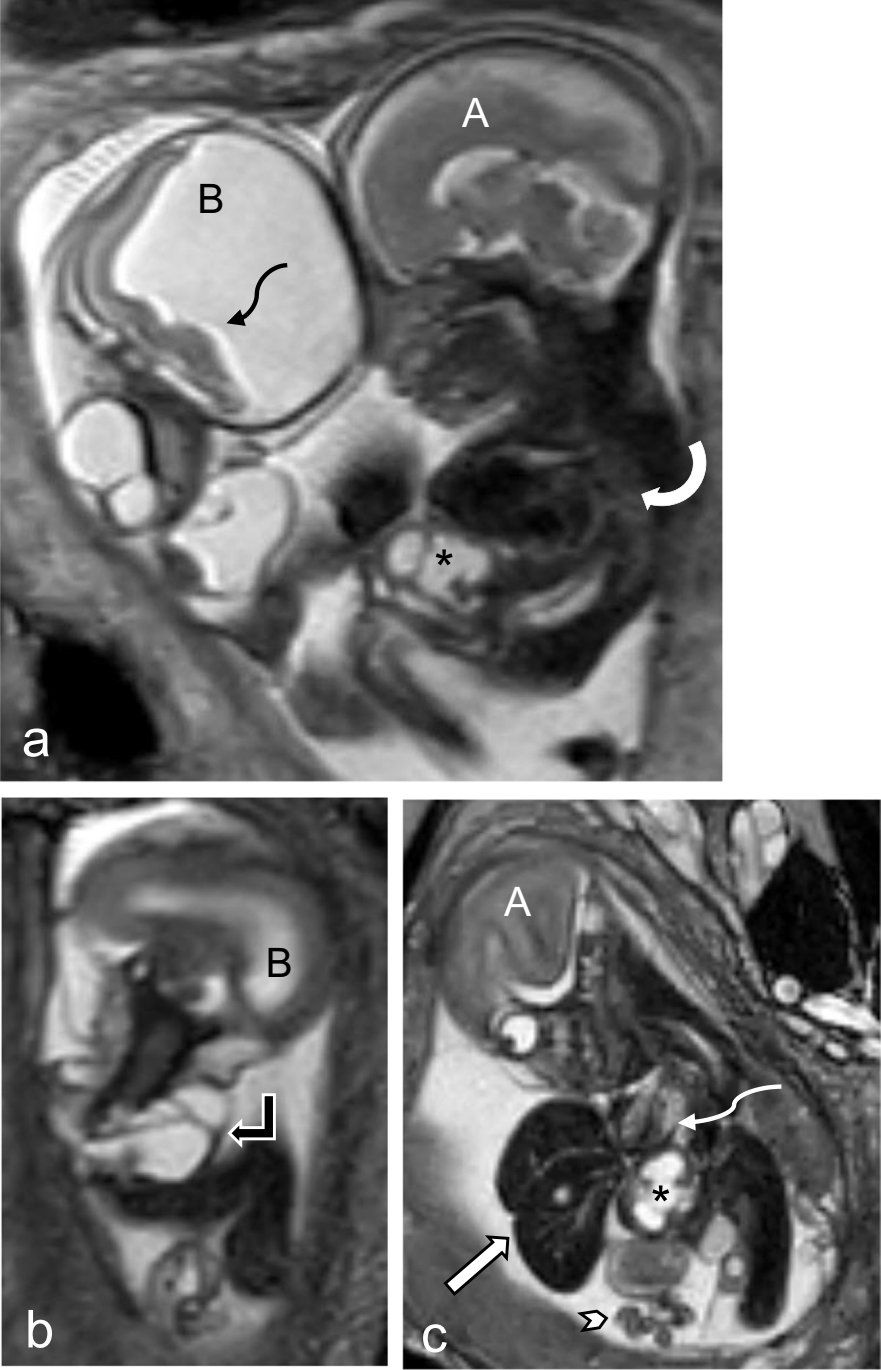
Fetal MRI of craniopagus at gestational age 24 weeks, 6 days, showing dorsal cephalic union, in addition to substantial individual congenital anomalies. This pair was joined only by a scalp skin bridge (confirmed at birth). **a** T2-weighted SSH TSE sagittal image to twin A’s brain shows separate intracranial compartments for twin A and twin B. Twin B demonstrates alobar holoprosencephaly (*curvy black arrow*), while twin A demonstrates a hypoplastic chest (*curved white arrow*) with partially imaged thoracoabdominoschisis and pelvicaliectasis (*black asterisk*). **b** BTFE image coronal to twin B’s body shows a terminal myelocystocele (*black right-angle arrow*). Twin B has a nonviable appearance. Twin B’s chest is small without candidates for heart or lung tissue. Additionally, there was no identified liver, spleen, kidneys, or bladder in twin B. **c** BTFE sagittal to twin A’s body shows thoracoabdominoschisis with liver evisceration (*white arrow*), bowel evisceration (*white arrowhead*), mild ectopia cordis (*curvy white arrow*), and pelvicaliectasis (*black asterisk*). Twin A also had evisceration of spleen and stomach with scoliosis and absence of the lumbosacral vertebral bodies with overall findings consistent with limb body wall complex. *BTFE*, balanced turbo-field echo; *SSH TSE*, single-shot turbo spin-echo

Craniopagus are an uncommon conjoined twin morphological variant that are dorsally conjoined at the head at locations other than the face and foramen magnum, although the union usually involves the forebrain region and very rarely the cerebellum [20] (Fig. 7). Craniopagus have separate trunks with four arms and four legs [19]. Whereas other conjoined twin morphological variants commonly have a mirror-image type of orientation with respect to one another, the orientation and angulation of craniopagus union are variable [20]. The degree of tissue sharing at the head is also variable and may include shared skull, dural venous sinuses, and brain parenchyma [19]. One review of craniopagus found brain parenchymal fusion in approximately 30% of pairs [42]. Craniopagus may be divided into “total” and “partial” subgroups. Total craniopagus have a continuous shared calvarium, often with sharing of the dural venous sinuses, whereas the degree of fusion of partial craniopagus is less extensive [42]. If there is no dural separation between the brains, a shared venous sinus may course circumferentially around the edge of the calvarial fusion. Other dural venous sinus variants observed in craniopagus include a shared superior sagittal sinus, two interconnected superior sagittal sinuses, or venous sinus lakes [43]. With rare exceptions, the cerebral arteries are typically separate [42–45]. Craniopagus have been reported to have cardiovascular, genitourinary, and craniofacial anomalies [43].

##### Pygopagus

**Fig. 8:**
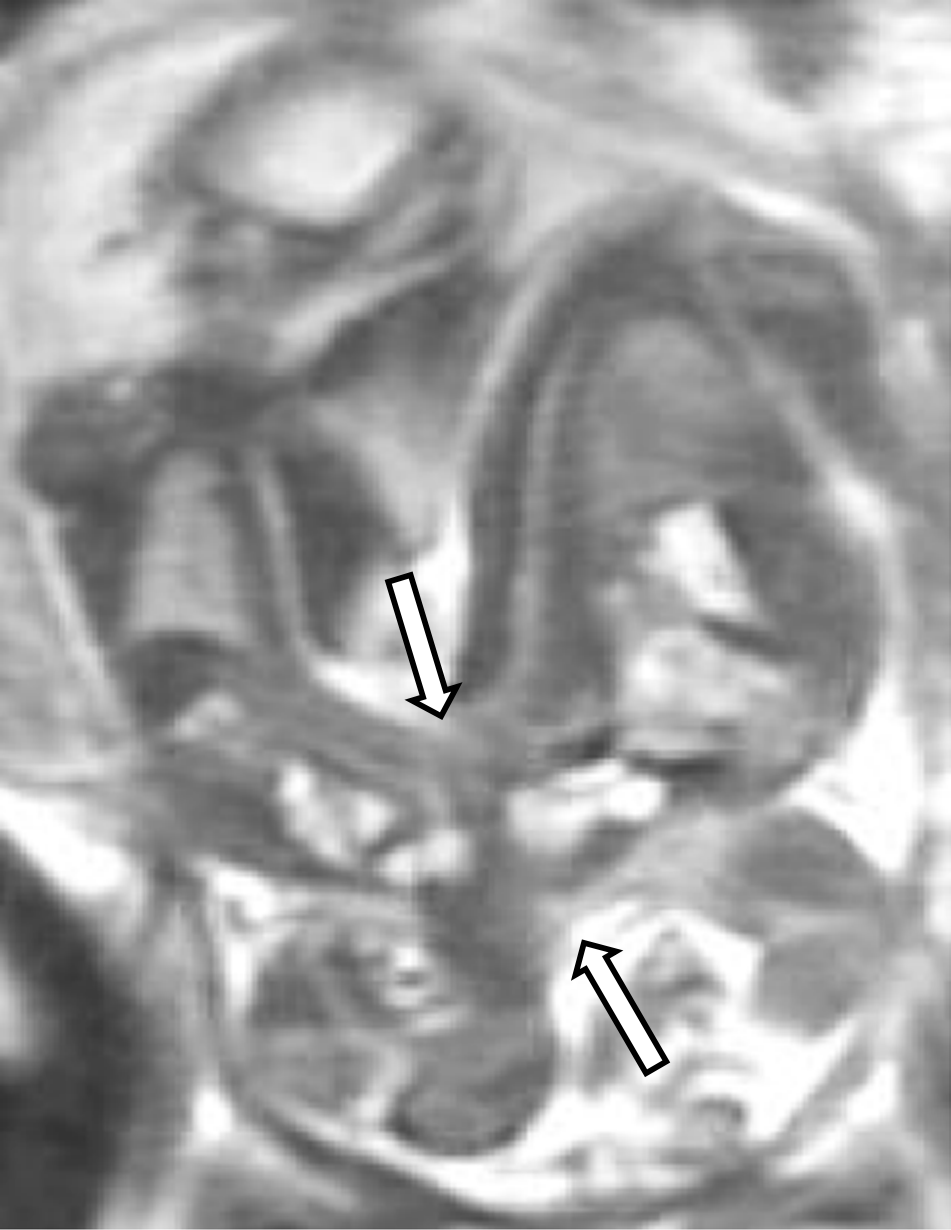
T2-weighted fetal MRI of pygopagus showing dorsal fusion in the sacrococcygeal region (*white arrows*). Image provided courtesy of Dorothy Bulas

Pygopagus are dorsally united in the sacrococcygeal and perineal locations, and each twin has their own paired arms and legs [14, 19] (Fig. 8). About a third of pygopagus have spinal dural fusion, commonly accompanied by spinal cord fusion [41]. In one series, 80% had coccyx and thecal sac fusion, 16% had lumbar vertebral fusion, and 11% had neural tissue fusion, although crossed neural tissue was not observed [46]. About 50% of pairs will have a shared rectum and/or anus, whereas 15% will have a shared genitourinary system [25, 29, 46]. One-half of pygopagus may have anomalies remote from their fused anatomy, including cardiac, renal, gastrointestinal, and extremity anomalies [46]. The presence of hemivertebrae may produce scoliosis [40]. Pygopagus may also have subluxed or dislocated hips or congenital foot deformities [14, 47].

##### Rachipagus

**Fig. 9:**
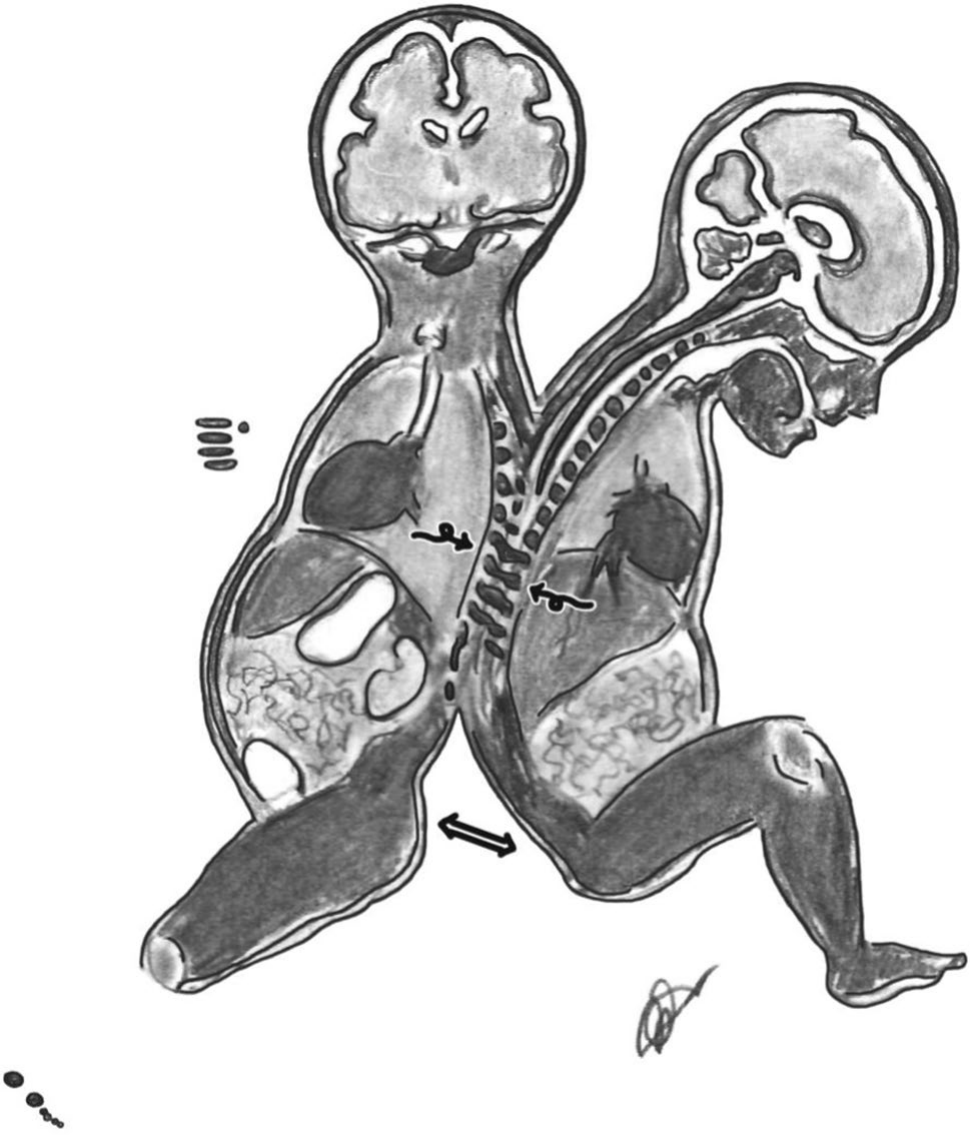
Artist’s representation of fetal MRI appearance of rachipagus conjoined twins. The twins are joined dorsally at the spine (*curvy black arrows*) above the level of the sacrum (*double-headed arrow*). The spinal canal and cord are shared, but out of the simulated imaging plane. Original artwork by Julie Walcutt, edited with Photoshop 2025

Rachipagus are extremely rare; Spencer reported only a single example of a typical symmetric rachipagus conjoined twin pair in a series of 1200 conjoined twin reports. Rachipagus are dorsally united above the level of the sacrum, which may extend as cephalad as the occiput (Fig. 9). Shared posterior spinal elements create a shared spinal canal [48].

#### Special variant: diamniotic conjoined twins

**Fig. 10:**
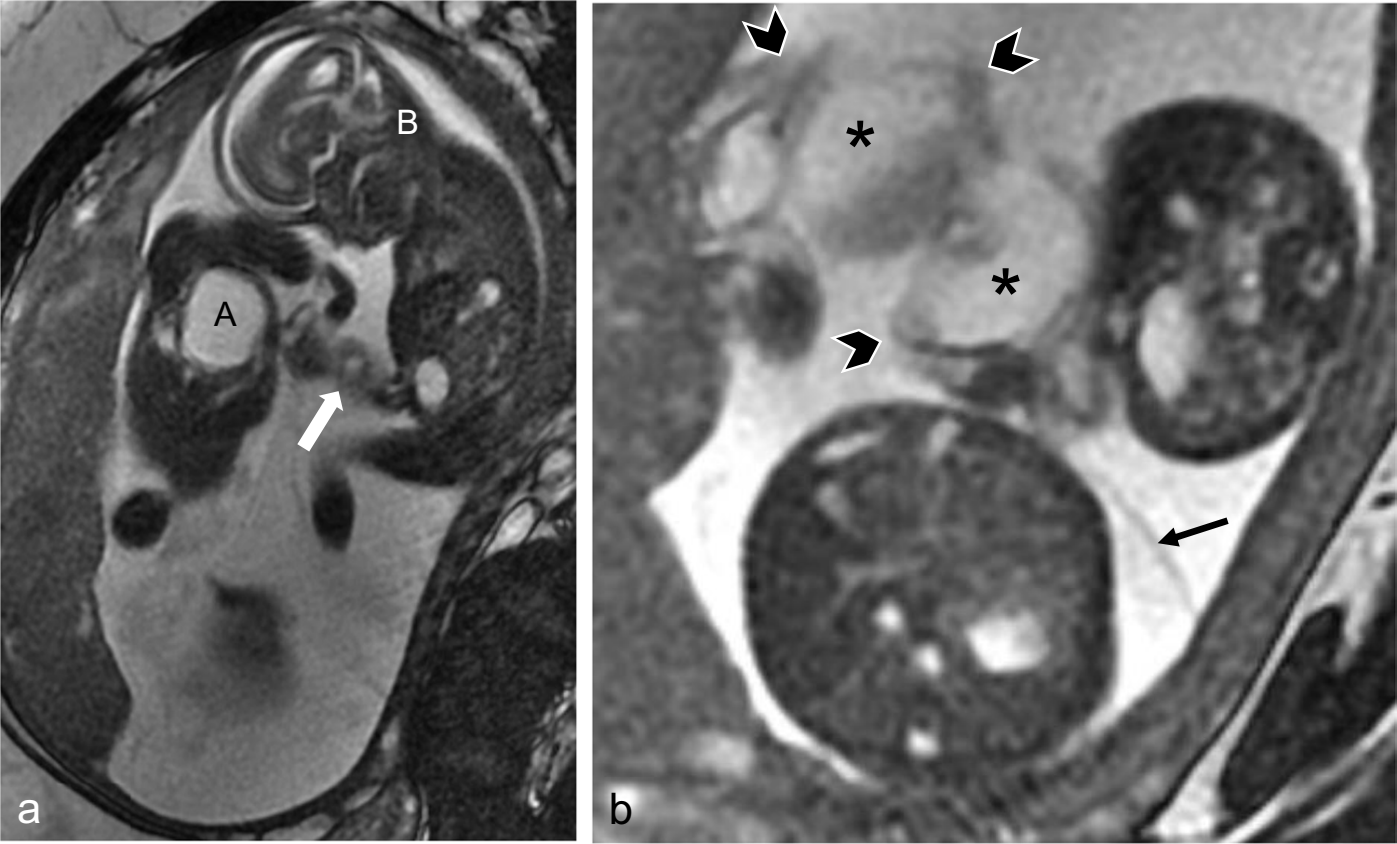
Fetal MRI of diamniotic minimally conjoined omphalopagus at gestational age 19 weeks. **a** Two-dimensional FIESTA sagittal to twin B shows herniated bowel (*white arrow*) extending across the fused tissue to twin A. **b** Two-dimensional FIESTA axial to the twins’ separate abdomens shows the umbilical cords (*black arrowheads*) inserting onto the shared allantoic cysts (*black asterisks*). The intertwin membrane of this diamniotic pregnancy is visible (*black arrow*). *FIESTA*, fast imaging employing steady-state acquisition

Although most conjoined twins are monoamniotic, diamniotic conjoined twins have been observed in the form of minimally conjoined omphalopagus. These twins are united at the level of a shared allantoic cyst or cysts onto which the umbilical cords insert (Fig. 10). These twins typically demonstrate intestinal fusion, and additional congenital anomalies are common, including anal atresia and cloacal anomalies [49]. Diamniotic craniopagus have also been reported [20].

## Conjoined twin congenital anomalies

Conjoined twins often demonstrate congenital anomalies that are superimposed upon their fusion, with those of particular importance to each morphological variant discussed above. These structural anomalies may be discordant between the twins [41]. The right-sided twin of ventrolaterally conjoined pairs is more likely to demonstrate abnormalities, including dextrocardia, with varying degrees of angulation along the plane of fusion allowing for distinction between the right- and left-sided twins [19, 21, 50]. The right-sided twin is also more likely to have fewer than three umbilical vessels in those pairs that have a shared umbilical cord [21]. The reported frequency of anomalies in different noncardiac organs in conjoined twins is detailed in Table 2. In one database review, two or more major anomalies were observed in 27% of cases [7]. Interestingly, the first English-language description of esophageal atresia was detailed in a 1670 case report of a thoracopagus pair [51].

**Table 2:** Frequency of congenital anomalies in conjoined twins

Organ system or type of anomaly	Reported frequency
Musculoskeletal or limb anomalies	10–36%
Genitourinary anomalies	13–20%
Gastrointestinal anomalies or atresias	10–17%
Central nervous system anomalies or neural tube defects	7–10%
Facial clefts	10–14%
Abdominal wall defects	13–25%
Congenital diaphragmatic hernia	5–6%

[2, 7, 8]

Congenital heart disease is more common among all morphological variants of conjoined twins compared to the general population, with cardiac anomalies seen in at least one twin in 66% of pairs [14, 22] (Fig. 6, part d). Cardiac anomalies are more common in ventrolaterally conjoined compared to dorsally conjoined twins (100% of thoracopagus, 49% of parapagus, 32% of omphalopagus and cephalopagus, and 10% of ischiopagus, compared to less than 10% of craniopagus, pygopagus, and rachipagus) [22]. It has been proposed that an inadequate number of umbilical vessels, more commonly seen in the right-sided twin of ventrolaterally conjoined pairs, interferes with cardiac development in this twin, producing defects [21]. For both twins, if the hearts are separate, the cardiac anomalies observed are of the types seen in singletons. Complex multi-chamber anomalies and vascular interconnections may be seen in pairs with conjoined hearts [20]. Hearts with ventricular fusion typically also have atrial fusion [52].

## Conjoined twin separation and survival

Not all conjoined twin pairs will be candidates for separation based upon the viability of the twins and the potential mortality and disability anticipated with their separation [11, 34]. Table 3 summarizes the anatomic features of conjoined twinning that likely preclude surgical separation, and Table 4 summarizes the potential for viability and separation of conjoined twin pairs according to their morphological variant.

**Table 3:** Anatomic features of conjoined twinning that likely preclude surgical separation

Organ or organ system	Anatomic feature
Heart^a^	Ventricular or atrial fusion Congenital heart disease of separate hearts
Liver^b^	Shared hepatic venous drainage Single portal vein or triad
Central nervous system^c^	Extensive brain parenchymal fusion Extensive dural venous sinus sharing Extensive spinal cord fusion in parapagus or rachipagus
All^d^	Extensive body and head fusion seen in cephalopagus, dicephalic or diprosopic parapagus Severe shared or individual congenital anomalies Significant chromosomal anomalies

^a^[27, 59]

^b^[17]

^c^[11, 14, 42]

^d^[3, 41]

**Table 4:** Conjoined twin viability and potential for separation by morphological variant

Morphological variant	Potential for separation	Viability/post separation survival
Thoracopagus^a^	Inversely related to degree of cardiac fusion Impossible in 80–90%	Determining overall separation and survival rates for thoracopagus is challenging due to lack of consistency in how these pairs have been categorized in the literature Typically die within weeks of birth if atrial and ventricular fusion is present
Omphalopagus^b^	Most likely variant to be separable due to lack of cardiac or major neurovascular fusions	~ 80% survive separation
Cephalopagus^c^	Inseparable	Nonviable
Ischiopagus^d^	Likely	60–80% survive separation
Parapagus^e^	Dicephalic and diprosopic parapagus not separable Separability of dithoracic parapagus dependent upon extent of organ sharing; extensive dorsal neurological fusion may be prohibitive	Poor prognosis for dicephalic and diprosopic parapagus
Craniopagus^f^	Dependent upon degree of intracranial fusion, separation not attempted in ~ 30%	Attempted separation resulted in survival of both twins in 46%, survival of one twin in 21%, and death of both twins in 4%
Pygopagus^g^	Likely	87% post separation survival rate
Rachipagus^h^	Unreported, potentially impossible	Unknown

^a^[8, 13, 14, 26, 52–54, 60–62]

^b^[4, 11, 41]

^c^[3, 14, 31, 41, 68]

^d^[4, 25, 57]

^e^[1, 11, 41]

^f^[43]

^g^[4, 46]

^h^[4, 41]

The substantial cardiac fusion seen in many thoracopagus and some parapagus, cerebral fusion in all cephalopagus and some craniopagus, and extensive body fusion of many parapagus and rachipagus preclude separation of these pairs [17, 53]. Conjoined twins with significant individual or shared anomalies or chromosomal variants may be inseparable [54].

Sometimes emergent separation is attempted if one twin dies or is so ill they may precipitate the death of the co-twin, if a treatable surgical emergency exists in one or both twins, or if the fused tissue itself is damaged (such as in the case of a ruptured omphalocele), although survival rates with emergency separation are less than 30% [55–57]. Ideally, medically stable pairs are separated electively after a months-long period of growth and careful multi-disciplinary team planning and rehearsal [34, 53]. With elective separation, approximately 80% of individuals may survive [55].

**Fig. 11:**
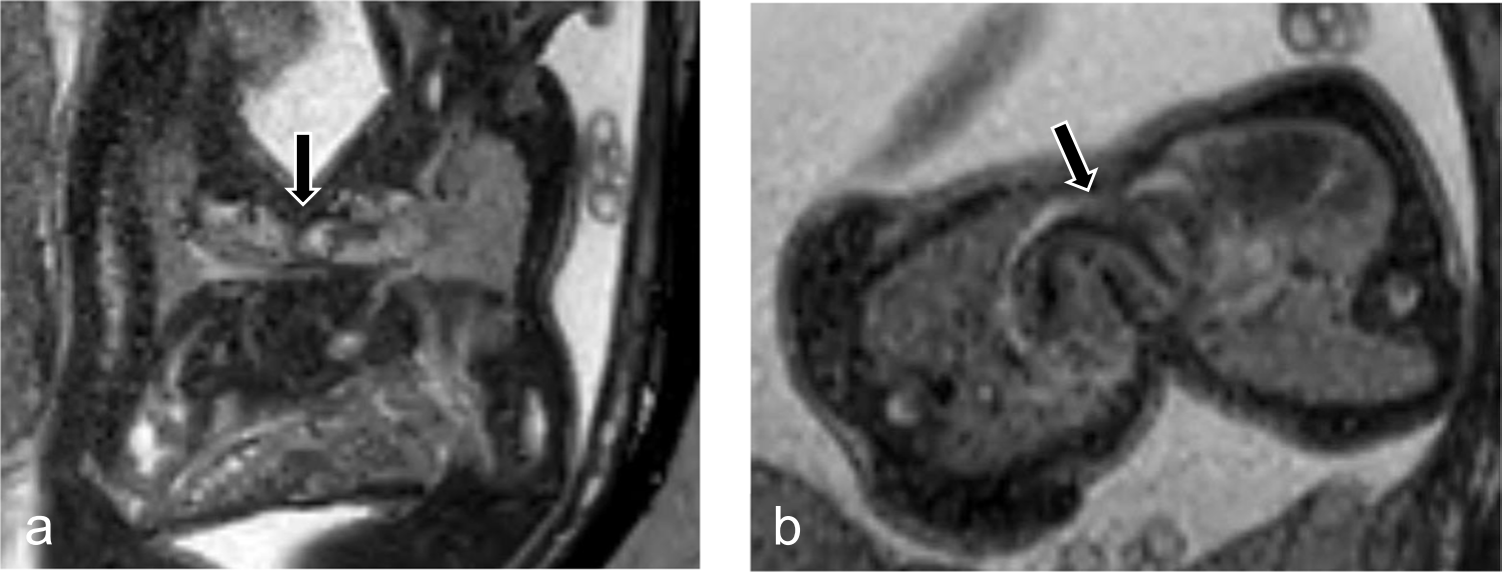
Fetal MRI of thoracopagus at gestational age 24 weeks, 6 days. **a** BTFE sagittal and **b** BTFE axial to the conjoined torso both show a shared, complex multichambered heart (*black arrows*). *BTFE*, balanced turbo-field echo

**Fig. 12:**
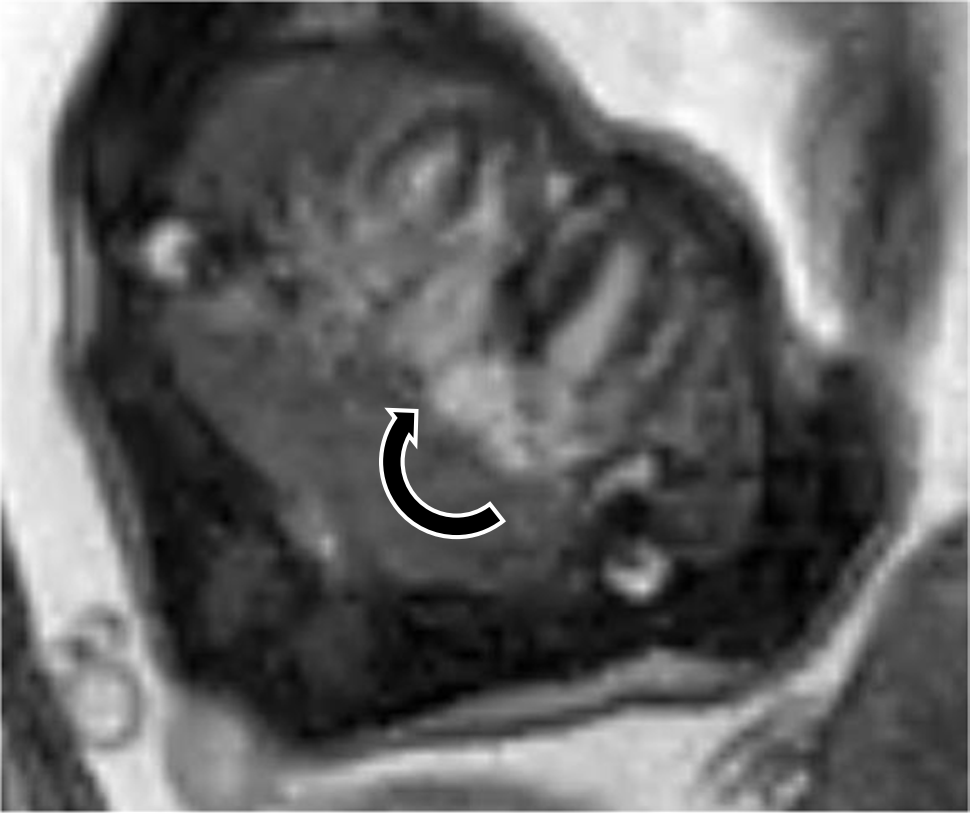
Fetal MRI (BTFE) of dicephalic parapagus at gestational age 25 weeks, 1 day, axial to the conjoined chest shows fused atria (*curved black arrow*). *BTFE*, balanced turbo-field echo

The heart is one of the most important considerations for elective separation of conjoined twins. Echocardiography reliably evaluates cardiac fusion and anomalies in conjoined twins and, for thoracopagus with ventrally fused chests, may be easier to perform prenatally when amniotic fluid can augment the sonographic windows that may be limited postnatally due to the shared chest wall configuration and lung aeration [14, 26, 58]. Survival in the setting of conjoined twinning and congenital heart disease is poor with or without separation [59]. Conjoined twins with atrial and ventricular fusion have complex superimposed cardiac anomalies and are not considered separable, typically dying within weeks of birth [8, 13, 26, 52–54, 60–62] (Fig. 11). Even with only atrial fusion, survival after separation has been poor [26, 63] (Fig. 12). However, it may be possible to separate tubular atrial connections, minimal ventricular non-luminal myocardial fusion, and fused conduction systems [22, 32, 60, 64]. A thoracopagus pair with a coronary sinus connection has been successfully separated [28]. A systematic review of conjoined twin (categorized as thoracopagus by the authors) outcomes following separation reported survival rates of 50% for pairs with a shared atrioventricular- or ventricular-level fibromuscular bridge, 32% for pairs with a shared atrial channel, 10% for pairs with atrial fusion, and 6% for pairs with ventricular fusion or a communicating ventricular channel. The same study reported a 50% survival rate for pairs with large vessel conjunction in the setting of either two separate hearts or one heart perfusing an acardiac twin [27]. Respiratory pathophysiology plays a substantial role in the mortality of conjoined twins with cardiac fusion [52]. Pericardial fusion with separate hearts does not preclude separation and does not significantly affect survival [26].

Attempts to separate conjoined twins with a shared heart by sacrificing one twin have typically resulted in the death of both, although exceptions have been reported [25, 29, 38, 60]. Separation of a thoracopagus pair in which a single normal heart was perfusing the nonviable co-twin via umbilical cord flow reversal (a case of conjoined twin reversed arterial perfusion sequence) resulted in one survivor [65]. In another case of ventrally conjoined twins, emergency separation performed at birth allowed survival of one twin with a structurally normal, functionally failing heart, who was perfusing a co-twin with a separate rudimentary heart via a communicating vessel [62].

**Fig. 13:**
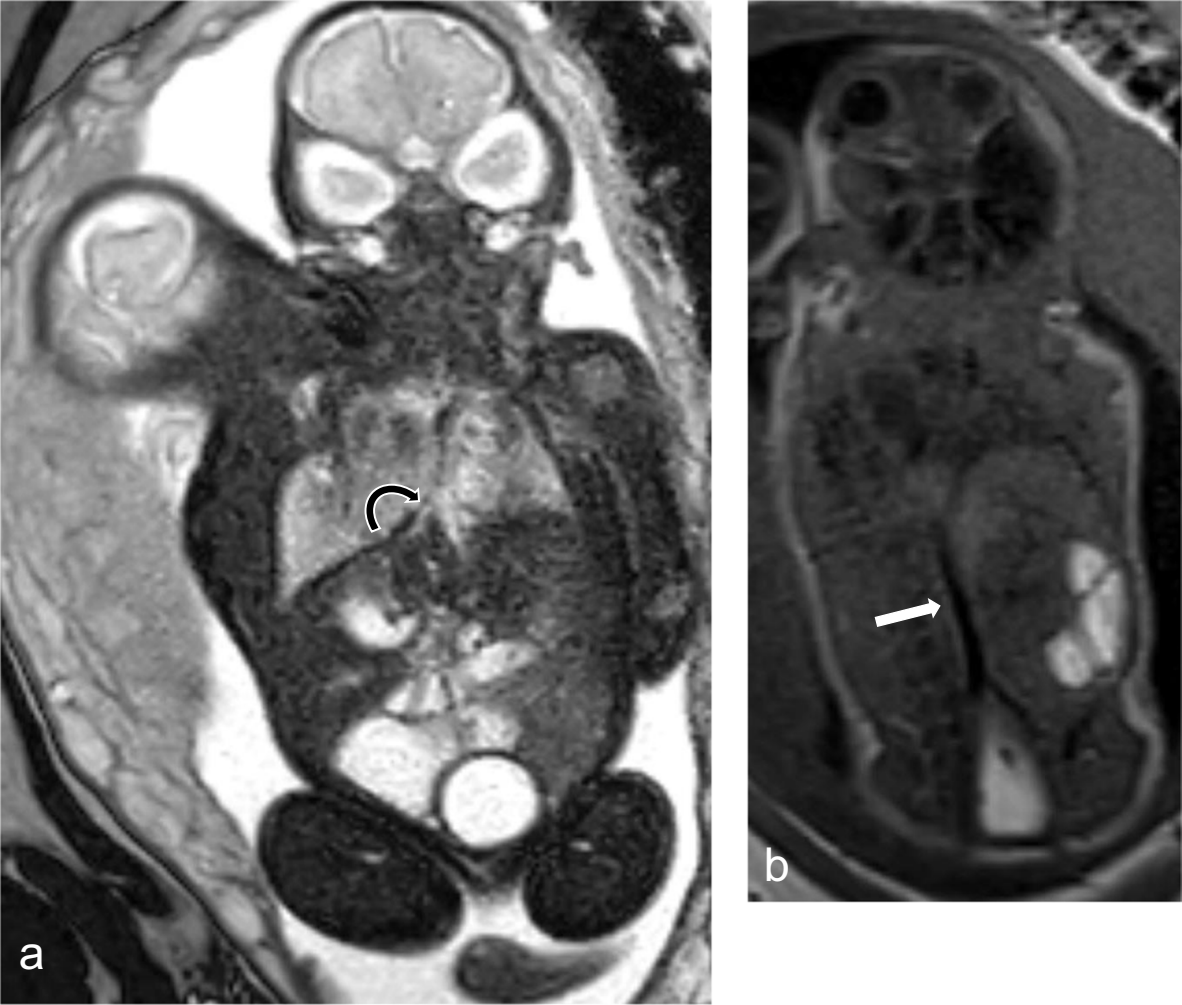
Fetal MRI of dicephalic parapagus at gestational age 32 weeks, 1 day (same case as depicted in Fig. 6), showing vascular sharing. **a** BTFE image coronal to the conjoined torso shows the hepatic veins of the shared liver draining into the heart of the left-sided twin (*curved black arrow*). **b** T1-weighted image coronal to the conjoined torso shows a flow void in the shared abdominal aorta (*white arrow*), which appeared to be a continuation of the left-sided twin’s thoracic aorta. *BTFE*, balanced turbo-field echo

The biliary anatomy and hepatic vasculature may also determine whether a conjoined twin pair may be separable. Hepatobiliary sharing, which may range from simple shared hepatic parenchyma to varying degrees and complexity of extrahepatic biliary fusion, is generally amenable to separation but may require Roux-en-Y reconstruction [54]. Biliary fusion is uncommon in the setting of separate gallbladders but has been reported [24, 32]. Operative reconstruction becomes very challenging if there is only one common bile duct and may not be reasonably possible if there is only one portal vein or portal triad [17]. Both twins must have their own hepatic venous drainage to allow separation [14, 17, 24, 38] (Fig. 13).

Among the morphological variants with cephalic fusion (cephalopagus, diprosopic parapagus, and craniopagus), only craniopagus are potentially separable. However, if substantial brain parenchymal fusion is present in these twins, separation may not be possible [14]. Knowledge of the dural venous sinus anatomy is also crucial, given the potential for substantial blood loss during separation [43]. Additionally, preferential venous drainage into one twin may produce important hemodynamic consequences [44]. Some advocate staging of procedures for craniopagus separation to allow venous collateral development [44]. A 1987 review of craniopagus separations found an inverse correlation between the degree of venous sharing and survival; however, the same study found that shared brain tissue was more commonly seen in cases with poor neurological outcomes, rather than appearing to impact survival [45]. A more recent study found surgical outcomes were worse for total craniopagus compared with partial craniopagus, with mortality rates of 48% and 14%, respectively [42]. Another study found that craniopagus with vertical (rather than angular) conjunction were more likely to be successfully separated, possibly due to less frequent posterior fossa fusion, reducing the technical complexity of the separation [43].

Even with spinal cord fusion, pygopagus have been separated without substantial neurological deficits; separation reduces the obstacles to ambulation of the conjoined state and of the cord tethering that results from spinal cord sharing [66]. Separation of symmetric rachipagus has not been reported, and those with extensive spinal cord fusion would likely be inseparable [20].

Other features of conjoined twinning, while not precluding separation, add to its complexity. A single shared anus may be divided between the twins (possibly following anal dilation) with acceptable fecal continence if there is adequate sphincter tissue. Higher levels of rectal fusion are associated with poorer functional outcomes in pygopagus [46]. In ischiopagus, pelvic osteotomies are typically required to reconfigure the large conjoined pelvis into two pelvic rings and facilitate ventral abdominopelvic wall closure [34, 57]. For male ischiopagus lacking duplicated external genitalia, the shared penis may have additional corpora allowing division and penile reconstruction for each twin [17, 67]. In all morphological variants, separation and reconstruction may require tissue expanders, flap creation, negative pressure therapy, mesh, or use of a nonfunctional limb to cover the resultant large soft tissue defects [33, 34, 67].

## Conclusion

Prenatal imaging plays a critical role in the care of conjoined twins. The superb structural detail and large field-of-view imaging offered by fetal MRI is well equipped to delineate the complex conjoined anatomy and possible superimposed anomalies that will determine each pair’s prognosis and is an excellent adjunct to fetal ultrasound. With a thorough understanding of the conjoined twin morphological variants, common anomalies, and anatomical implications for survival and separation, fetal imagers will be best prepared to communicate this essential information to families and clinicians navigating prenatal care decisions and planning for multi-disciplinary postnatal care.

## Supplementary Information

Below is the link to the electronic supplementary material.

Supplementary file1 (DOCX 18 KB)

## Data Availability

No datasets were generated or analysed during the current study.
