# Supplementary material for: Fetal MRI findings in conjoined twin pregnancies
Source: Pediatr Radiol. 2025 Aug 15;56(8):1757–75. doi: 10.1007/s00247-025-06323-1 (PMC13407584; doi:10.1007/s00247-025-06323-1)
Supplement: Supplementary file 1 — Supplementary file1 (DOCX 18 KB) [file 247_2025_6323_MOESM1_ESM.docx]

**Supplementary Table 1** Conjoined twin fetal MRI dictation template

| CLINICAL HISTORY: []^a^.  COMPARISON: [None OR obstetric ultrasound {date}].  PROCEDURE COMMENTS: MRI was performed without contrast to evaluate the uterus and fetuses. |
| --- |
| FINDINGS: The assigned gestational age of the pregnancy is [] weeks, [] days, by [last menstrual period, early ultrasound] with an estimated date of delivery of []. |
| Maternal findings: None. |
| Findings of pregnancy:  Pregnancy: [Monoamniotic], monochorionic conjoined twins.  Size concordance: [Concordant, discordant].  Placental location: [Anterior, posterior, fundal.]  Placental previa: [No, yes].  Placental signal: [Homogeneous, heterogeneous.]  Placental thickness: [] cm.  Uterus: Normal.  Cervix: Closed.  Cervical length: [] cm. |
| Fetal findings:  Site of union: [Ventral, lateral, dorsal.]  Morphological conjoined twin subtype: [Thoracopagus, omphalopagus, cephalopagus, ischiopagus, parapagus, craniopagus, pygopagus, rachipagus.]  Twin assignments: Both twins are in [vertex, breech, transverse] presentation. Twin A is located in the [left, right, anterior, posterior, upper, lower] portion of the uterus. Twin B is located in the [left, right, anterior, posterior, upper, lower] portion of the uterus.  Number of umbilical cords: [One, two].  Number of vessels in shared umbilical cord: [].  Shared umbilical cord insertion site: [Paracentral, marginal, membranous], series [], image #[].  *If separate umbilical cords, separately describe for Twin A and Twin B.* |
| Site of union: *Example descriptions of the conjoined twin morphological subtypes as below (example descriptions may differ from actual observations).* |
| **Thoracopagus:** The twins are ventrally joined from the chest through the upper abdomen to the level of the shared umbilicus. Twin A has [2] arms and [2] legs. Twin B has [2] arms and [2] legs. There is a single shared multichambered heart that is [centrally located within the shared chest OR eccentrically located more within the chest of Twin {A, B}]. *Describe great vessels.* Evaluation of the cardiovascular anatomy limited by technique. Please refer to fetal echocardiogram for details. *Describe any other shared chest anatomy.* There is a large, shared liver with [2] gallbladders. Bowel loops extend across the plane of union in the shared upper abdomen. |
| **Omphalopagus:** The twins are ventrally joined from the [chest, lower chest] through the upper abdomen to the level of the shared umbilicus. Twin A has [2] arms and [2] legs. Twin B has [2] arms and [2] legs. Each twin has its own separate heart. *Describe any other shared chest anatomy.* There is a large, shared liver with [2] gallbladders. Bowel loops extend across the plane of union in the shared upper abdomen. |
| **Cephalopagus:** The twins are ventrally joined from the head through the upper abdomen to the level of the shared umbilicus. Twin A has [2] arms and [2] legs. Twin B has [2] arms and [2] legs. There is a single shared head. [There appears to be a single set of facial structures on one side of the shared head, *describe*, OR Two sets of facial structures are arranged side-by-side on one side of the shared head, *describe symmetry/asymmetry, etc*., OR Two sets of facial structures are seen, arranged 180 degrees apart on opposite sides of the shared head, *describe symmetry/asymmetry, etc*.] Within the shared head, there is a shared brain, demonstrating cerebral fusion but separation of the hindbrain with two separate cerebellums and brainstems. The cervical spinal canals and cords are separate. *Describe other shared or separate anatomy of the head, face, and neck, including ears, eyes, maxilla, mandible, and pharynx.* [Within the shared chest, there is a single shared multichambered heart OR There are separate hearts for each twin.] *Describe great vessels.* Evaluation of the cardiovascular anatomy limited by technique. Please refer to fetal echocardiogram for details. *Describe any other shared chest anatomy.* There is a large, shared liver with [2] gallbladders. Bowel loops extend across the plane of union in the shared upper abdomen. |
| **Ischiopagus, *if aligned face-to-face*:** The twins are joined ventrally from the shared umbilicus through the pelvis. Twin A has [2] arms and [2] legs. Twin B has [2] arms and [2] legs. The chests are separate with each twin having its own separate heart. The pelvis is conjoined, *describe*. [There is one shared urinary bladder OR There are two bladders, *describe spatial arrangement* *of urinary bladders*.] *Describe renal drainage to bladder(s) if possible. Describe separation or sharing of genitalia and rectums and arrangement.*  **Ischiopagus, *if aligned end-to-end*:** The twins are united in an end-to-end fashion at the pelvis with a shared umbilicus. Twin A has [2] arms and [2] legs. Twin B has [2] arms and [2] legs. The chests are separate with each twin having its own separate heart. The pelvis is conjoined. The sacrums, spines, and spinal cords are separate. There are two pubic symphyses, each positioned laterally between the twins, with each twin contributing one pubic ramus to each symphysis, completing a single, large, conjoined pelvic ring. At each pubic symphysis, there is a set of genitalia shared by both twins and flanked on either side by two legs, one belonging to each twin. [There is one shared urinary bladder OR There are two bladders, *describe spatial arrangement of urinary bladders*.] *Describe renal drainage to bladder(s), if possible. Describe separation or sharing of rectums and arrangement.* |
| **Parapagus, *if dithoracic*:** The twins are joined laterally, side-by-side, at the level of the abdomen and pelvis with two separate chests. There is a single shared umbilicus. Twin A has [2] arms. Twin B has [2] arms. [There are two shared legs OR There are 3 legs, one belonging to each twin with a third leg shared between them, *describe*.] There are two separate hearts, one in each chest. *Describe great vessels.* There is a large, shared liver with [2] gallbladders. There are two stomachs. Bowel loops extend across the plane of union in the shared peritoneal cavity. There is a single pair of kidneys in the shared abdomen. There is a single urinary bladder in the shared pelvis. The rectum and external genitalia are shared. [There is one sacrum, *describe any spinal fusion*, OR There are two sacrums with separate spines.]  **Parapagus, *if dicephalic*:** The twins are joined laterally, side-by-side, with a shared chest, abdomen, and pelvis. There is a single shared umbilicus. [There are two arms, one belonging to each twin, OR There are 3 arms, one belonging to each twin with a third arm shared between them, *describe*.] [There are two shared legs OR There are 3 legs, one belonging to each twin with a third leg shared between them, *describe*.] [Within the shared chest, there is a single shared multichambered heart OR There are separate hearts for each twin, *describe any asymmetry*, OR There are side-by-side hearts with *describe level of cardiac fusion*.] *Describe great vessels.* Evaluation of the cardiovascular anatomy limited by technique. Please refer to fetal echocardiogram for details. *Describe number of lungs and other shared chest anatomy.* There is a large, shared liver with [2] gallbladders. There are two stomachs. Bowel loops extend across the plane of union in the shared peritoneal cavity. There is a single pair of kidneys in the shared abdomen. There is a single urinary bladder in the shared pelvis. The rectum and external genitalia are shared. [There is one sacrum, *describe any spinal fusion*, OR There are two sacrums with separate spines.]  **Parapagus, *if diprosopic*:** The twins are joined laterally, side-by-side, with a shared head, chest, abdomen, and pelvis. There is a single shared umbilicus. There are two arms and two legs. Within the shared head, there are two sets of side-by-side facial structures, *describe*. All other structures in the head, chest, abdomen, and pelvis are shared, with detailed assessment as below. *Utilize standardized organ reporting as below for unshared structures.* |
| **Craniopagus:** The twins are joined dorsally, at [*describe head location*]. *Describe orientation (twins face the same or in different directions, vertical or angular union). Describe what tissues are shared (skin, calvarium, dural venous sinuses, brain parenchyma).* All other structures are separate. |
| **Pygopagus:** The twins are joined dorsally in the sacrococcygeal region. [The thecal sacs are separate OR The thecal sacs are joined, *describe neural element separation or sharing if possible*.] [The rectums are separate OR The rectum is shared, *describe degree/length of hindgut sharing.*] |
| **Rachipagus:** The twins are joined dorsally through the spine, extending from the [] to [] levels. *Describe neural element separation or sharing, if possible.* |
| *General template for fetal anatomy. Utilize for unshared structures only, repeating for each twin, noting abnormal positioning or other anomalies:*  TWIN [A, B]:  Brain:  Calvarial shape: Normal.  Frontal-occipital diameter: [] mm.  Bone biparietal diameter: [] mm.  Cerebral biparietal diameter: [] mm.  Sulcation: Normal.  Ventricles: Normal, dilated (*provide measurements)*.  Cavum septum pellucidum: Normal.  Germinal matrix: Normal.  Cerebral venous sinuses: Normal, dilated (*describe)*.  Corpus callosum: Normal.  Brain stem morphology: Normal.  Anterior-posterior pons diameter: [] mm.  Cerebellum: Normal.  Transverse cerebellar diameter: [] mm.  Cerebellar vermis: Normal.  Vermis foliation: Normal.  Vermis craniocaudal x anterior-posterior diameter: [] x [] mm.  Craniocervical junction: Normal.  Extra-axial fluid spaces: [Normal, increased, decreased.]  Face/neck:  Oropharynx/cervical airway: Normal.  Facial profile: Normal.  Eyes: Normal.  Interocular distance: [] mm.  Binocular distance: [] mm.  Lips and nose: Normal.  Mandible: Normal.  Ears: Normal.  Nuchal fold: Normal.  Spine:  Vertebrae: Normal.  Spinal canal: Normal.  Spinal cord: Normal.  Conus position: Normal.  Chest:  Chest size/shape: Normal.  Airway: Normal.  Mediastinum/thymus: Normal.  Lungs: Normal.  Heart: Subjectively normal size. Evaluation of the cardiovascular anatomy limited by technique. Please refer to fetal echocardiogram for details.  Aorta: Normal.  Abdomen/pelvis:  Abdominal wall/soft tissues: Normal.  Situs: Normal.  Inferior vena cava/aorta: Normal.  Liver: Normal.  Gallbladder: Normal.  Spleen: Normal.  Stomach: Normal.  Small bowel: Normal.  Colon/rectum: Normal.  Rectal cul-de-sac length: [] mm.  Right kidney: Normal.  Left kidney: Normal.  Urinary bladder: Normal.  Genitalia: [Female, male, not visualized.]  Extremities: Normal. Note: Detailed evaluation of the extremities is limited on fetal MRI. |

^a^Brackets indicate fill-in fields or pick lists with suggested options listed within. Template modified from template used at Cincinnati Children’s Hospital Medical Center for reporting of twin pregnancy fetal MRI.
